# Temporal response of an injectable calcium phosphate material in a critical size defect

**DOI:** 10.1186/s13018-021-02651-8

**Published:** 2021-08-13

**Authors:** Jacob T. Landeck, William R. Walsh, Rema A. Oliver, Tian Wang, Mallory R. Gordon, Edward Ahn, Colin D. White

**Affiliations:** 1 Department of Biochemistry and Molecular Pharmacology, University of Massachusetts Medical School, Worcester, MA 01605 USA; 2 Graduate School of Biomedical Sciences, University of Massachusetts Medical School, Worcester, MA 01605 USA; 3 Surgical & Orthopaedic Research Laboratories (SORL), Prince of Wales Clinical School, Level 1 Clinical Sciences Building, Prince of Wales Hospital, UNSW Sydney, Sydney, Australia; 4 Anika Therapeutics Inc., Bedford, MA USA; 5 X-Factor Capital, Dover, MA 02030 USA; 6 Vertex Pharmaceuticals, 50 Northern Ave, Boston, MA 02210 USA

**Keywords:** Calcium phosphate, Cement, Bone healing, Defect, Rabbit

## Abstract

**Background:**

Calcium phosphate-based bone graft substitutes are used to facilitate healing in bony defects caused by trauma or created during surgery. Here, we present an injectable calcium phosphate-based bone void filler that has been purposefully formulated with hyaluronic acid to offer a longer working time for ease of injection into bony defects that are difficult to access during minimally invasive surgery.

**Methods:**

The bone substitute material deliverability and physical properties were characterized, and in vivo response was evaluated in a critical size distal femur defect in skeletally mature rabbits to 26 weeks. The interface with the host bone, implant degradation, and resorption were assessed with time.

**Results:**

The calcium phosphate bone substitute material could be injected as a paste within the working time window of 7–18 min, and then self-cured at body temperature within 10 min. The material reached a maximum ultimate compressive strength of 8.20 ± 0.95 MPa, similar to trabecular bone. The material was found to be biocompatible and osteoconductive in vivo out to 26 weeks, with new bone formation and normal bone architecture observed at 6 weeks, as demonstrated by histological evaluation, microcomputed tomography, and radiographic evaluation.

**Conclusions:**

These findings show that the material properties and performance are well suited for minimally invasive percutaneous delivery applications.

## Background

Calcium phosphates either in granular, putty, or injectable forms are osteoconductive biomaterials with a long preclinical and clinical history as bone graft substitutes in a variety of clinical applications. Considering the clinical indication, different forms of calcium phosphate may be preferred from a surgical perspective to facilitate surgical implantation as well as minimize soft tissue exposure. The percutaneous use of calcium phosphate bone substitute materials has a variety of clinical applications including bone voids such as cysts, and osteoarthritis-related bone marrow oedema lesions, and insufficiency fractures [1–4]. The improved surgical handling of an injectable material provides surgeons with an easy and efficient manner to deliver these materials into bony voids or deficiencies to augment the local bony environment. However, there are few truly injectable calcium phosphates commercially available that can be internally delivered into closed structures [5].

Here, we present an injectable calcium phosphate (CaP) bone graft substitute material containing hyaluronic acid that self-hardens post deployment at body temperature to poorly crystalline apatite, like the inorganic constituent of bone. The ease of injectability allows for its implantation into bony defects that are difficult to access, without consequence to curing time post deployment or mechanical properties, for application in osseous defects for minimally invasive surgery. Hyaluronic acid is a naturally occurring polysaccharide in the human body and is one of the largest components of the extracellular matrix. For this bone substitute material, hyaluronic acid enhances flowability and imparts cohesion to the paste. These are important parameters to improve deliverability into voids of irregular geometry and interdigitate with the trabecular bone architecture without excessive pressure. Hyaluronic acid has also been shown to improve osteoblast precursor differentiation, indicative of osteogenesis, on CaP materials [6]. Enhanced apatite deposition has also been demonstrated with use of hyaluronic acid [7]. Further, the cell binding ability of hyaluronic acid has been shown to play a role in osteoclast-mediated bone resorption [8].

A complete review of the historical developments related to CaP bone substitute materials are addressed by authors in previous publications [9–12]. The current study reports the physical properties, mechanical properties, and in vivo response of a new CaP bone material substitute meant with the inclusion of hyaluronic acid. A critical size defect model [13] was used to evaluate the in vivo response out to 26 weeks to assess the interface with the host bone as well as implant degradation and resorption in vivo.

## Methods

### Physical and mechanical characterization

#### Handling parameters

The device was comprised of a separate 4 mL aqueous solution containing hyaluronic acid, citric acid, and sodium phosphate dibasic and a 4 g powder containing α-tricalcium phosphate, calcium carbonate, and monocalcium phosphate. The powder and liquid were manually mixed to homogeneity in a closed system using an integrated mixing device over the course of 1 min. The paste was transferred to 1 cc syringes then fitted to a cannula. Cannulas of 15 Ga and 60 mm length dimensions and 13 Ga and 110 mm length dimensions were separately evaluated. Cannulas were inserted into an open cell rigid polyurethane foam (Open Cell Block 15 PCF, Saw Bones), submerged in 37 °C phosphate-buffered saline (PBS; pH 7.4). The working time was determined by the time interval that the combined 4 g and 4 mL could be extruded as a cohesive paste through the cannula using digital pressure for *n* = 4 samples. Injectability was defined as the paste ability to remain homogeneous under digital pressure during injection without phase separation for *n* = 4 samples. Setting was measured on the paste filled into cylindrical stainless-steel moulds (6-mm diameter and 12-mm height) submerged in 37 °C simulated body fluid (SBF), pH 7.4. Setting time was measured by placing a final Gillmore needle (453.6 g in weight and 1.06 mm in diameter) onto the surface of the hardened CaP cylindrical specimen for *n* = 6 samples.

#### Characterization

Cylindrical samples (6-mm diameter and 12-mm height) of the CaP material were prepared by injection into a stainless-steel mould submerged in PBS, pH 7.4, at 37 °C for compressive strength and dimensional stability. Compressive strength and dimensional stability samples were prepared for measurement over the course of the setting reaction in simulated body fluid (SBF), pH 7.4, at 37 °C. At each time point (24, 48, 96, 144, 192, 240, 288, 336, and 384 h), the mean ± standard deviation was obtained for *n* = 20 samples of each mass, length, diameter, and ultimate compressive strength (UCS). Samples were tested with a cross head speed of 0.5 in/min until fracture using an electromechanical testing machine (Chatillon TCM 200). Compressive strength was calculated using the maximum force and cross-sectional area of the sample.

### In vivo evaluation

The in vivo response of the injectable CaP material was evaluated in a critical size distal femur defect in skeletally mature rabbits using an established surgical model and experimental endpoints [13]. Empty defects served as negative controls and corticocancellous autograft harvested from the iliac crest as a positive control. Time points of 6, 12, 18, and 26 weeks study design and endpoint allocation are summarized in Table 1. The sample sizes were calculated based on previous experience with this model [13]. The critical defect nature of the model was validated out to 18 weeks using empty defects.

**Table 1:** In vivo study design

	Endpoints									
	Paraffin histology					PMMA histology/histomorphometry				
**Sites at weeks:**	0	6	12	18	26	0	6	12	18	26
Empty defect	NA	10	10	6	0	NA	6	6	3	0
Autograft	NA	10	10	6	3	NA	6	6	3	0
CaP material	NA	10	10	6	6	NA	6	6	6	6

Study design reporting the number of implantation sites at each time point and the corresponding endpoints (NA = not applicable)

Institutional ethical clearance (UNSW ACEC 17/39A) was obtained prior to using an established model and experimental endpoints [13]. One hundred and eight skeletally mature female New Zealand (NZ) white rabbits were enrolled in the study with a mean weight of 3.8 kg (± 0.3 kg). Skeletal maturity was confirmed prior to enrolling in surgery based on radiographic screening and confirmation of growth plate closure of the tibial tuberosity fibula and distal femur. Lateral radiographs were taken (Poskom, Model PXP 60HF, Image Metrix, Sydney, Australia) with digital plates (Agfa). DICOM Works (ezDICOM medical viewer, copyright 2002) was used to evaluate the data. Growth plate closure was defined as a complete bony bridge and no evidence of radiolucency.

Animals were acclimatized for 7 days prior to surgery and housed in deep litter on floor pens. Animals were health checked and microchipped for identification. Animals were weighed prior to surgery and weekly thereafter throughout the study. The surgical procedure [13] began with sedation via a mixture of midazolam (0.3–0.5 mg/kg) and buprenorphine (0.03–0.05 mg/kg) intramuscularly using a 26G needle. Anaesthesia was induced and maintained using isoflurane and oxygen inhalation during surgery. A range of isoflurane between 1 and 3% along with oxygen (2 l per minute) was used. The animals were monitored for changes in vital signs (e.g. breathing and heart rate) during surgery as well as the response to pain to control the level of anaesthesia. Eye reflex and colour of mucous membranes was observed as well as oxygen levels monitored to ensure an appropriate level of anaesthesia during surgery.

**Fig. 1:**
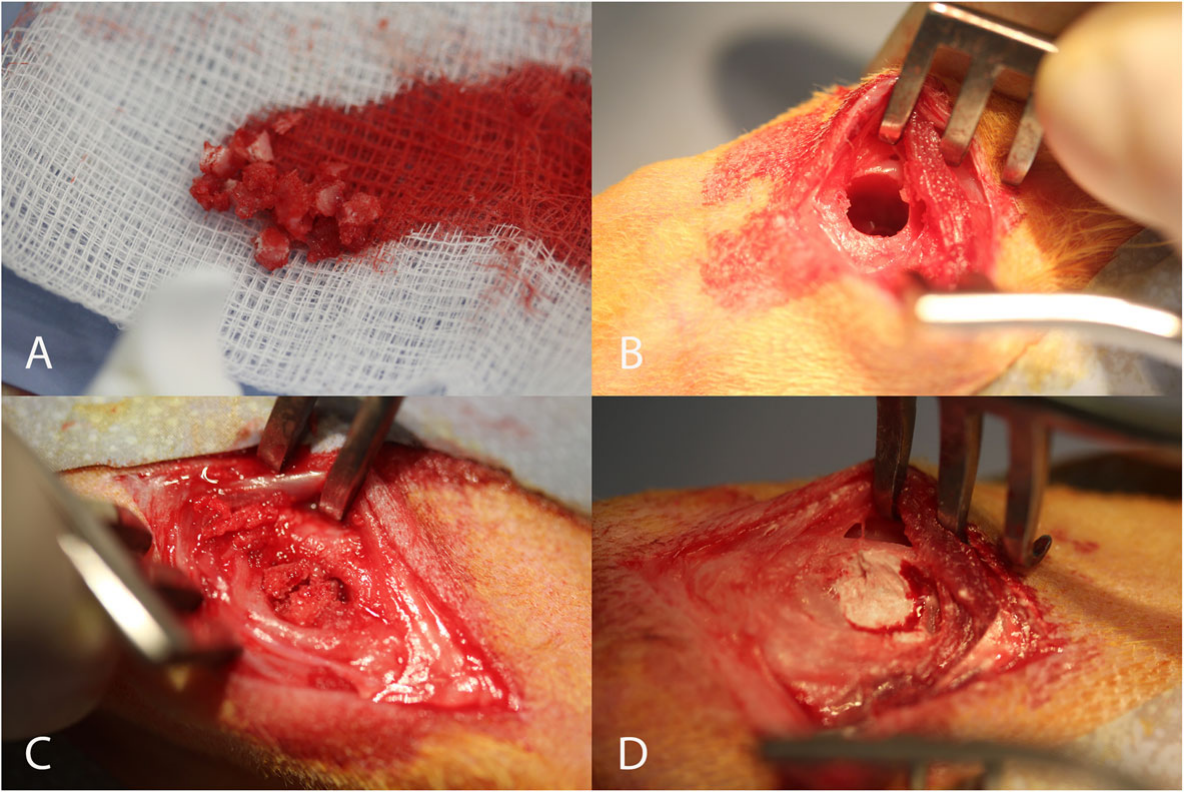
Surgery images showing autograft harvested from the iliac wing (**A**), defect (**B**), placing autograft in the defect (**C**), and the final CaP material implantation (**D**)

Corticocancellous autograft when required by the study design was harvested from the right iliac crest using a rongeur [14]. Bilateral critical defects (6-mm in diameter and 10-mm deep) were created in the cancellous bone of the medial distal femur [13]. A 1-cm skin incision was made to visualize medial collateral ligament (MCL) and identify the medial epicondyle. The defects were prepared with a pneumatic drill under saline irrigation to minimize thermal damage with a 4.5 mm 3 fluted pyramid tip drill (Surgibit, Orthopedic Innovations, Collaroy, NSW Australia) to avoid skiving and create a pilot hole followed by a 6-mm drill to a depth of 10 mm. The base of the defect was squared off with a 6 mm flat end mill. The CaP material was prepared as per manufacturer’s recommended instructions, described in handling parameters above, and carefully placed into the defects and filled to the height of the cortex (Fig. 1). Autograft (approximately 0.3 cc) was placed into the defect for the positive control group or the defect was left empty for the negative control group. The skin was closed using 3-0 Dexon (Davis & Geck, North Ryde, NSW). Animals were given post-operative analgesia (Temgesic, 1 ml subcutaneously) and returned to their holding cages. The animals were free to mobilize and weight-bear immediately post-operatively as tolerated.

At the designated time points, animals were weighed, scanned to confirm identification number, and anesthetized using isoflurane inhalation and euthanized by lethal injection of Lethabarb (Virbac Australia Pty Ltd, Milperra, NSW 2214) via cardiac injection. The right and left femora were harvested and photographed using a digital camera. The general integrity of the skin incision was noted along with the macroscopic reaction of the underlining subcutaneous tissues. This was noted as normal or abnormal in appearance.

The harvested femora were radiographed using Faxitron and high-resolution mammography film (settings 30 kV for 30 s) in the AP and lateral planes to evaluate for any bony abnormalities and assess radiographic evidence of implant resorption. Microcomputed tomography (μCT) was performed on all animals using an Inveon in vivo microcomputer tomography scanner (Siemens Medical, PA, USA) in order to obtain high resolution images of the implantation site at euthanization. The femora were scanned, and the raw images reconstructed to DICOM data using Siemens’s software (Inveon™ Research Workplace IRW). Images were examined in the axial, sagittal, and coronal planes to assess the healing at the implantation sites and examined for bony reactions and implant resorption versus time.

The distal femurs were fixed in 10% phosphate-buffered formalin at room temperature with gentle rotation on a Labtech rotating shaker, for a minimum of 96 h. The samples allocated for paraffin histology were decalcified in 10% formic acid -formalin used for ISO 10993-6 (2016) to evaluate the implant–host bone interface versus time. The decalcified samples were sectioned in the sagittal plane into 3 blocks from medial to lateral. The cut sections (~ 3 mm in thickness) were placed into embedding blocks for paraffin processing. Each paraffin block was sectioned (5 microns) using a Leica Microtome and placed on slides for haematoxylin and eosin (H&E) and tetrachrome staining.

Stained sections were examined under light microscopy using an Olympus Microscope with an Olympus DP72 high resolution video camera to capture images. The reviewer was blinded to time points and treatment groups. Histology was qualitatively assessed at each time point and a summary written. The stained slides were reviewed under low magnification to provide an overview of the section for documentation purpose using × 1.25 objective (scale bar = 1 mm). Specifically, the nature and extent of any tissue reaction observed was recorded as well as the presence and form of the implant.

The samples allocated for polymethymethacrylate (PMMA) (hard tissue) histology were dehydrated through a series of increasing concentrations of ethanol: 70–80–90–95–100% followed by MMA infiltration and final polymerization to PMMA. A Leica SP1600 saw-microtome (Leica, Nussloch, Germany) was used to cut 3 sections ~ 15 microns thick in the sagittal plane at three levels in the defect. The sections were etched with acidic ethanol (98 ml ethanol 96% and 2 mL HCl 37%) for 1 min and stained with methylene blue (Sigma, 1% in borax buffer (0.1 M) pH 8.5) for 1 min, followed by basic fuchsin (Sigma, 0.3% in water) for 1 min. Bone ongrowth to the surface of the materials was performed based on three sections per defect from the PMMA histology. The amount of bone ongrowth was evaluated with a qualitative grading scale blinded to material and time point: 0 = none, 1 = 1–25%, 2 = 25–50%, 3 = 51–75%, and 4 = 76–100% considering in vivo resorption profile of the materials and bone ongrowth was the only metric to assess. Data was presented as the mean of the qualitative grading versus time.

## Results

After mixing the powder and liquid phases to a paste for 1 min the CaP material continued to thicken and was injectable for a working time window of 7–18 min. Injectability, without phase separation, was possible through both a 15 Ga and 60 mm length cannula and a 13 Ga and 110 mm length cannula into an open cell rigid polyurethane foam, an alternative test medium for human cancellous bone submerged in 37 °C PBS. The setting time, the time it takes to reach a mechanical stability to withstand 5 MPa static pressure applied by the final Gillmor needle [9], was determined to be 10 min at body temperature.

The maximum ultimate compressive strength of 8.20 ± 0.95 MPa (average ± standard deviation) was measured at 192 h, after which the compressive strength remained stable throughout the course of the experiment (Table 2). Within the first 24 h, 53% of the ultimate compressive strength was reached, and by 48 h, 69% of the ultimate compressive strength was reached. Measurements of mass, diameter, and height showed no change, expansion, or shrinkage, over the course of the experiment demonstrating dimensional stability and insignificant loss through passive dissolution.

**Table 2:** Ultimate compressive strength of CaP material over time

Time (hours)	Ultimate compressive strength (MPa)	Standard deviation (MPa)
24	4.34	0.77
48	5.68	1.70
96	6.46	1.66
144	7.35	1.14
192	8.20	0.95
240	7.75	1.33
288	7.85	1.24
336	7.68	1.02
384	7.36	1.48

Surgery was completed without incident for all animals. The CaP material was easily implanted into the surgically repaired defect in the medial aspect of the distal femur (Fig. 1). No abnormalities were detected at the time of harvest with respect to skin incision healing or macroscopic inspection of the underlining subcutaneous tissues.

The Faxitron radiographs in the anteroposterior (AP) and lateral planes did not reveal any adverse reactions or bony abnormalities at any time point. The radiographs revealed well-placed defects with no evidence of infection or adverse reaction to the implanted materials based on radiographic appearance of the adjacent host bone or within the defect. The implanted CaP material was visible in the radiographs at all time points and did not display any significant evidence of resorption radiographically. The empty defects appeared empty in the lateral views at all time points. The autograft appeared visible in the lateral views and evidence of radiographic healing in this group was able to be discerned based on the Faxitron radiographs.

**Fig. 2:**
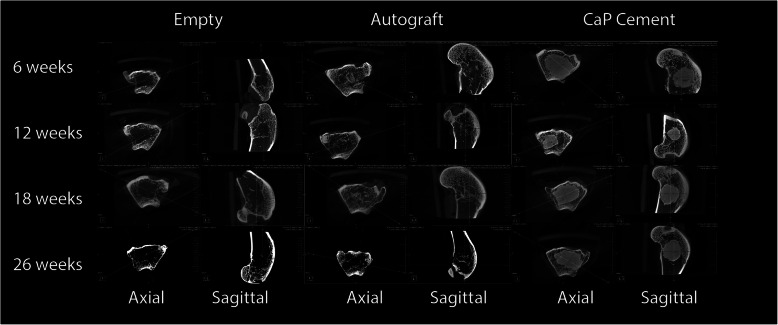
Microcomputed tomography overviews in the axial and sagittal planes for empty, autograft, and CaP material filled defects versus time. The empty defects remained empty reflecting the critical nature of the model. The autograft remodelled in the defect while the CaP material remained present with direct bone ongrowth as well as interdigitation with the host bone

Microcomputed tomography (Fig. 2) scanning revealed similar findings to the Faxitron radiographs. No adverse reactions were noted at the surgical sites in any animal at any time point in this study based. No evidence of infection or adverse reaction to the implanted CaP material of the adjacent host bone or within the defect. No graft resorption was observed out to 18 weeks, whereas some evidence of resorption for the CaP material was noted by 26 weeks, although this was minimal. The empty defects remained empty while the autograft treated defects progressed with time in terms of healing based on microcomputed tomography.

**Fig. 3:**
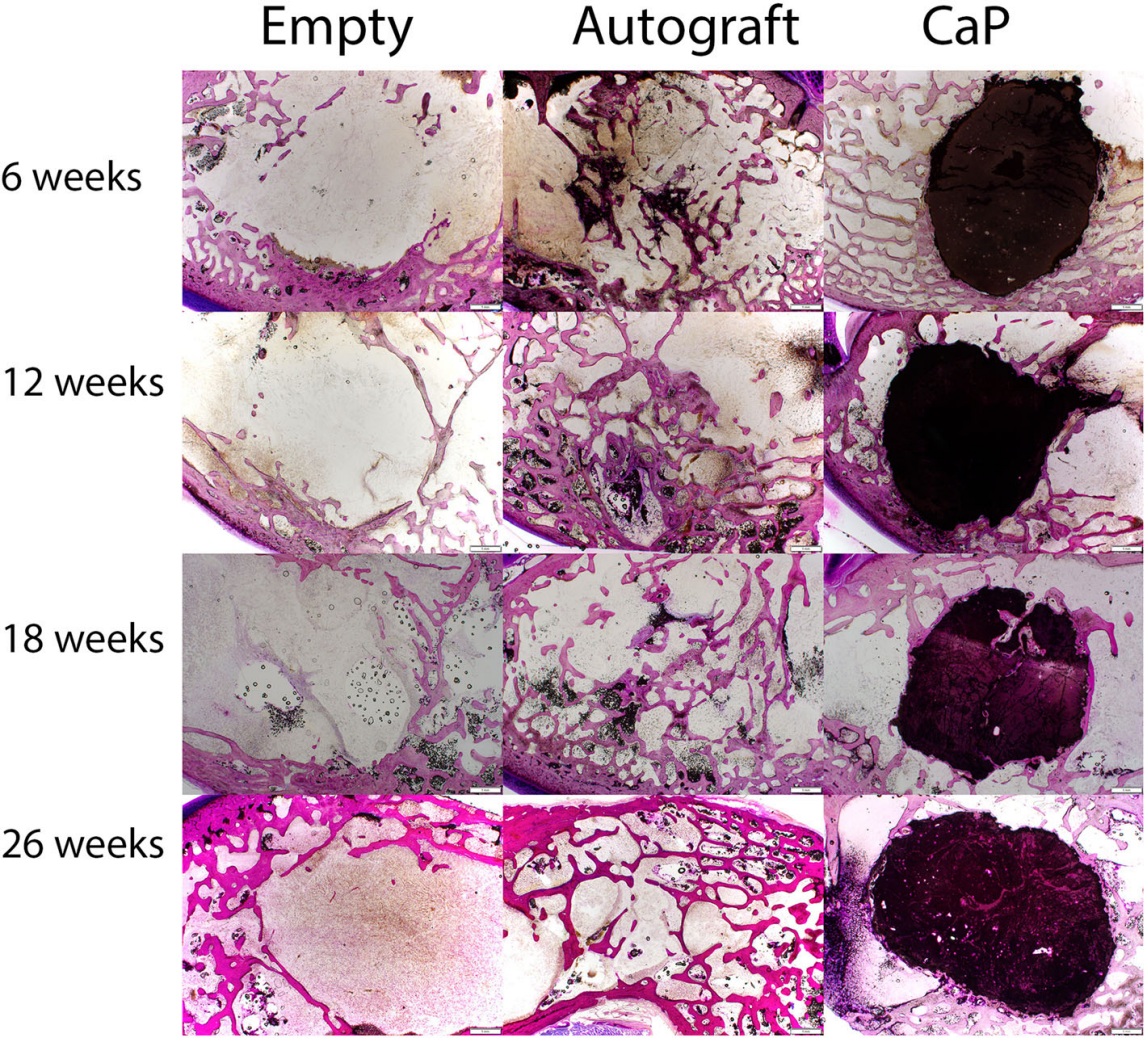
PMMA histology overviews in the sagittal plane for empty, autograft, and CaP material filled defects versus time. The empty defects remained empty reflecting the critical nature of the model. The autograft remodelled with time while the CaP material demonstrated direct bone ongrowth as well as interdigitation with the host bone

**Fig. 4:**
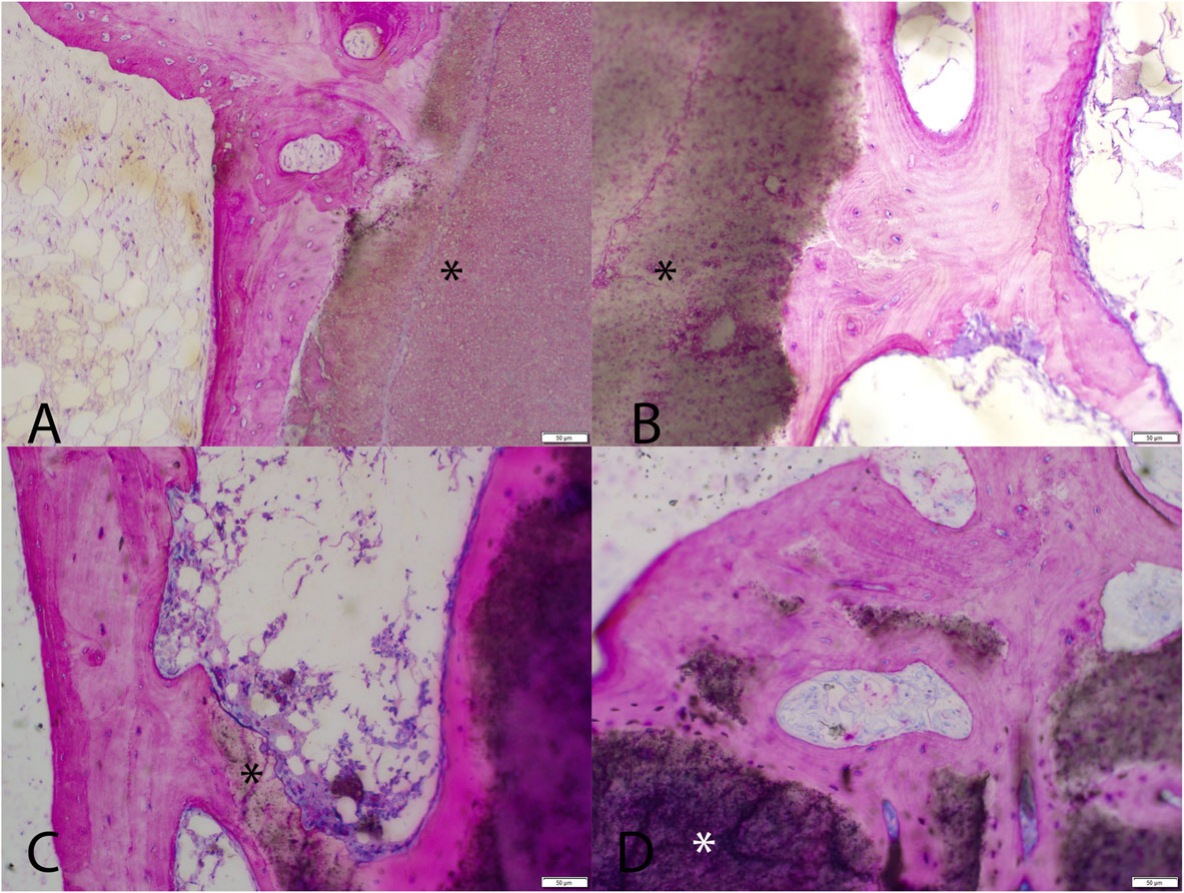
High magnification PMMA histology at the CaP material (*) bone interface at 6, 12, 18, and 26 weeks (**A**–**D**) demonstrating a direct interface between the host bone and the CaP material at all time points. Normal marrow spaces are present in the adjacent host bone. No adverse local reactions were noted

**Fig. 5:**
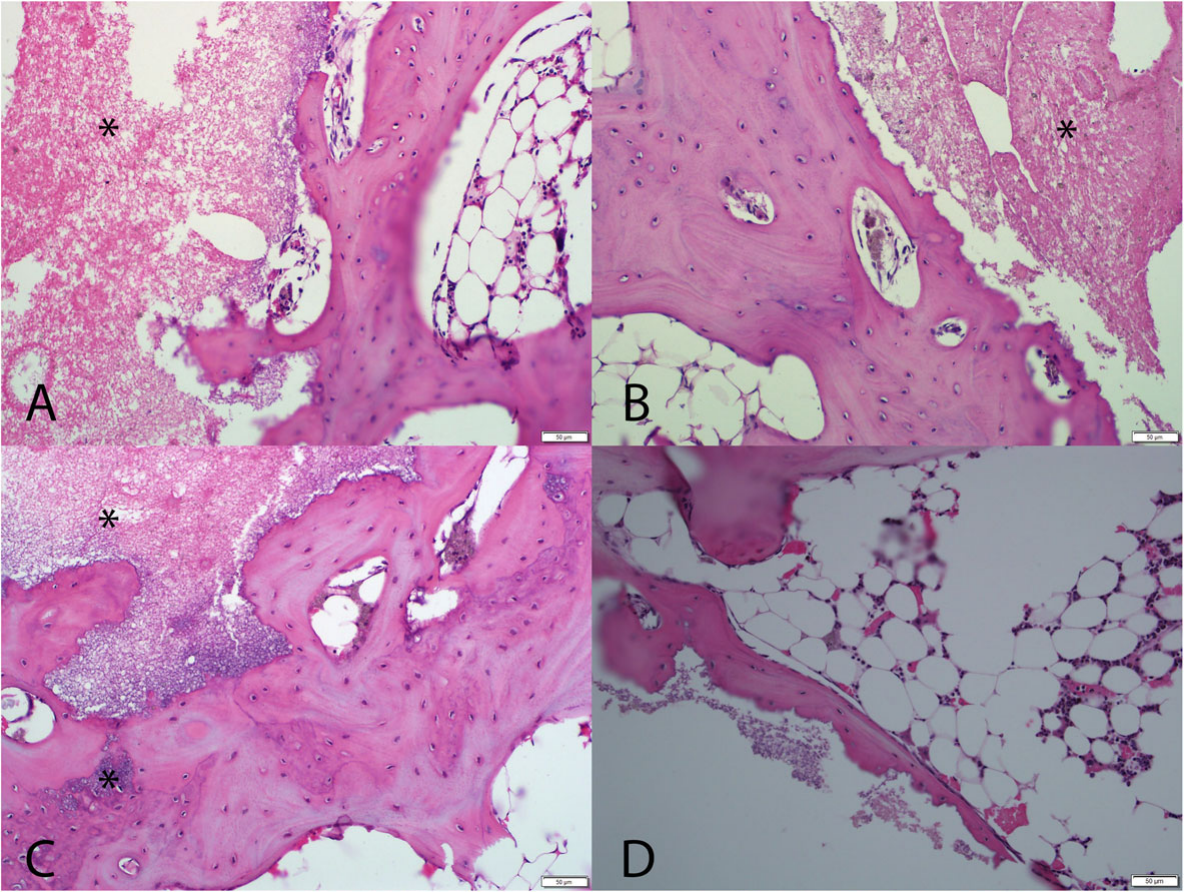
High magnification paraffin histology at the CaP material (*) bone interface at 6, 12, 18, and 26 weeks (**A**–**D**) demonstrating a direct interface between the host bone and the CaP material at all time points. Normal marrow spaces are present in the adjacent host bone. No adverse local reactions were noted

Consistent with the microcomputed tomography, the Paraffin and PMMA histology for each defect site was carefully sectioned moving from the medial aspect deeper into the defect to provide a comprehensive overview of the reaction at the tissue and cell level based on histology. The low magnification PMMA histology (Fig. 3) was used to evaluate the healing response at the margins considering the lack of resorption with the CaP material. New bone formation on the surface of the CaP material without any intervening fibrous tissue was noted along with no evidence of resorption as early as 6 weeks (Fig. 4). The surface of the CaP material was covered with bone at all time points with a mean grade of 3.88 (± 0.27) at 6 weeks and grades of 4 thereafter at 12, 18, and 26 weeks. No adverse reactions were noted at the margin with the host bone in in terms of acute or inflammatory cellular responses or fibrous tissue versus time for the CaP Material (Fig. 5). The histology of the autograft treated defects demonstrated resorption and remodelling of the autograft that was used to fill the defect for both paraffin and PMMA histology. This progressed with time, with newly formed woven bone that remodelled over time.

## Discussion

The inclusion of hyaluronic acid, a naturally occurring polysaccharide distributed widely throughout the human body, provides several advantages to the use and performance of the CaP bone substitute. When mixed into a paste, the natural biopolymer reportedly forms a hydrated network with anionic carboxylate groups capable of chelating dissolved calcium ions and hydrogen bonding with protonated phosphate ions to form an organic-mineral interface [6, 15, 16]. The high affinity for calcium ions also regulates the mineralization of CaP materials by temporarily stabilizing early stage crystallization and preventing aggregation [6]. The resultant paste is cohesive and flowable, imparting the ability to fill voids of irregular geometry such as trabecular bone architecture, without excessive pressure. The inclusion of hyaluronic acid has also been reported to improve the anti-washout ability by the resultant interlocking hydroxyapatite crystal lattice [7], important to reduce the release or microparticles which could cause adverse reactions.

Once self-hardened, hyaluronic acid may improve the bone apposition to the surface of calcium phosphates [7], evaluated by the ability for apatite precipitation and crystallization on the material surface [17]. Increased apatite deposition has been demonstrated with hyaluronic acid, which is proposed to occur as a result of hyaluronic acid dissolution and release of bound calcium ions on the material’s surface [7]. For the bone graft stability in the implant site, high osseointegration is important and resorption typically occurs from the outside layer by layer [18]. These findings are consistent with the in vivo data presented in the current study using a critical size defect in cancellous bone of the skeletally mature NZ White Rabbit. The radiographic data (Faxitron and microcomputed tomography) and histology demonstrated the formation of a bony interface between the material and the host without any intervening fibrous tissue layer. Resorption of the material was not observed during the time course of the current study while direct bone ongrowth was demonstrated as early as 6 weeks supporting the osteoconductive nature of this material.

For injectable calcium phosphates that self-harden in situ, the delivery procedure is dictated by the handling parameters. The injectable CaP material evaluated in the current study has a longer working time window (7–18 min) than many reportedly injectable materials [5]. While there is no agreement to the meaning of injectability [19], the force applied using a defined syringe geometry must be reasonably applied by an orthopaedic surgeon, reported as 100 N to 300 N [20]. Further, under the applied extrusion force, the paste should not lose homogeneity as a result of filter-pressing, which results in powder and liquid phase separation which has been reported for several commercially available CaP materials [5, 21]. Additionally, the setting time directly affects the clinical procedure by dictating the time post implantation when the surgeon can remove instruments and close the defect, without damaging the solidifying structure. The determined setting time of 10 min is like several commercial injectable materials [5].

The physical and mechanical properties examined in the current study reveal the CaP material hardens at body temperature and physiological pH with a mechanical strength appropriate for the biomechanical environment of cancellous or trabecular bone. The material requires sufficient mechanical strength to provide high resistance to deformation and allow the biological response to support bone remodelling. Ideally, the bone substitute material would have similar compressive strength to the surrounding bone to not alter the mechanical properties of the tissue [9]. However, strengths of calcium phosphates are often higher than natural bone, which can cause stress shielding to the surrounding bone. For this CaP bone substitute material, once fully hardened, the ultimate compressive strength measured in vitro was 8.20 ± 0.95 MPa. While the compressive strength of trabecular bone can vary widely [22, 23], this value is similar to reported compressive strengths of 2–12 MPa for cancellous bone [24].

The properties of this bone substitute material make it well suited for minimally invasive percutaneous delivery applications, such as for treating bone marrow oedema lesions or insufficiency fractures, because they require the ability to inject the self-hardening implant into a highly pressurized environment without damage to the tissue [25]. These surgical techniques require use of a minimal entry point for delivery of the bone substitute material to preserve integrity of the cortical bone.

This study is not without limitations. The study did not evaluate in vivo response to material prior to 6 weeks that may provide additional insight into the initial healing. The CaP material was limited to non-load bearing surgical sites due to mechanical strength required to satisfy those applications. Influence of resorption rate from the size of the implant, animal model, location of implantation, and disease state may also vary.

## Conclusion

The result of the current study demonstrates the material properties of this CaP bone substitute material and performance are well suited for minimally invasive percutaneous delivery applications.

## Data Availability

All the data pertaining to the present study have been included in this manuscript, and the authors are willing to share the raw data upon reasonable request to the corresponding author.

## Abbreviations

CaP: Calcium phosphate
PBS: Phosphate-buffered saline
SBF: Simulated body fluid
UCS: Ultimate compressive strength
PMMA: Polymethylmethacrylate
NA: Not applicable
MCL: Medial collateral ligament
μCT: Microcomputed tomography
H&E: Haematoxylin and eosin
AP: Anteroposterior
NZ: New Zealand
